# Supplementary material for: Methylation and Expression of FTO and PLAG1 Genes in Childhood Obesity: Insight into Anthropometric Parameters and Glucose–Lipid Metabolism
Source: Nutrients. 2021 May 15;13(5):1683. doi: 10.3390/nu13051683 (PMC8155878; doi:10.3390/nu13051683)
Supplement: Supplementary file 1 [file nutrients-13-01683-s001.zip › nutrients-1171431-supplementary.pdf]

nutrients

MDPI

Article

Methylation and expression of FTO and PLAG1 genes in childhood obesity: insight into anthropometric parameters and glucose-lipid metabolism

Wojciech Czogała [1], Małgorzata Czogała [1,2], Wojciech Strojny [1], Gracjan Wątor [3], Paweł Wołkow [3], Małgorzata Wójcik [4], Mirosław Bik Multanowski [5], Przemysław Tomasik [6], Andrzej Wędrychowicz [7], Wojciech Kowalczyk [8], Karol Miklusiak [8], Agnieszka Łazarczyk [8], Przemysław Hałubiec [8], and Szymon Skoczeń [1,2,*]

[1] Department of Pediatric Oncology and Hematology, University Children's Hospital of Krakow, Poland; czogala@tlen.pl (W.C.), Wojciech.strijny@mp.pl (W.S.)
[2] Department of Pediatric Oncology and Hematology, Faculty of Medicine, Jagiellonian University Medical College, Krakow, Poland; malgorzata.czogala@uj.edu.pl (M.C.), szymon.skoczen@uj.edu.pl (S.S.)
[3] Center for Medical Genomics – OMICRON, Jagiellonian University Medical College, Krakow, Poland; gracjan.wator@uj.edu.pl (G.W.), pawel.wolkow@uj.edu.pl (P.W.)
[4] Department of Pediatric and Adolescent Endocrinology, Faculty of Medicine, Jagiellonian University Medical College, Krakow, Poland; malgorzata.wojcik@uj.edu.pl
[5] Department of Medical Genetics, Faculty of Medicine, Jagiellonian University Medical College, Krakow, Poland; miroslaw.bik-multanowskitomasik@uj.edu.pl
[6] Department of Clinical Biochemistry, Faculty of Medicine, Jagiellonian University Medical College, Krakow, Poland; p.tomasik@uj.edu.pl
[7] Department of Pediatrics, Gastroenterology and Nutrition, Faculty of Medicine, Jagiellonian University Medical College, Krakow, Poland; andrzej.wedrychowicz@uj.edu.pl
[8] Student Scientific Group of Pediatric Oncology and Hematology, Jagiellonian University Medical College, Krakow, Poland; w.kowalczyk@student.uj.edu.pl (W.K.), karolmiklusiak@gmail.com (K.M.), agnieszka.lazarczyk@student.uj.edu.pl (A.L.), przemyslawhalubiec@gmail.com (P.H.)
* Correspondence: szymon.skoczen@uj.edu.pl; Tel.: +48503523785

Supplementary

Figure S1. Plots presenting the distribution of data of the studied parameters depending of the level of FTO expression (shown as log2 of the absolute value of expression for clarity of the plot). A. Insulin – OGTT 60 min (µIU/mL) B. Insulin – OGTT 120 min (µIU/mL) C. BF_kg D. BMI (kg/m2) E. HOMA-IR F. BMI percentile G. Waist circumference (cm) H. Waist circumference percentile.

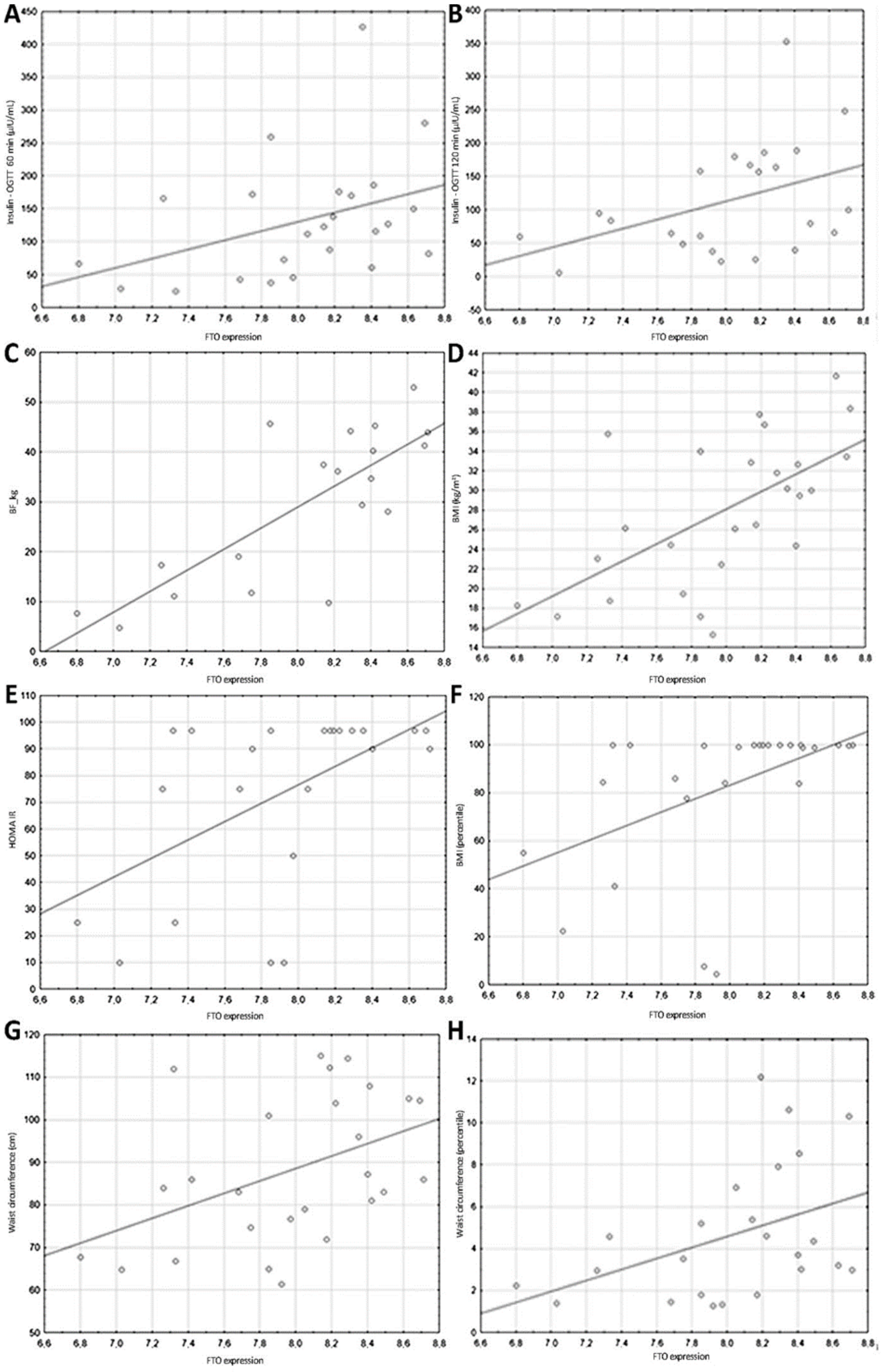

Table S1. Descriptive statistics of the children's age

Table S2. Correlation results of blood pressure with FTO gene methylation, expression and PLAG1 gene expression.

Group	Age mean	Age SD	Age Q25	Age median	Age Q75
**Obese children**	13.8	3.0	12.8	14.7	15.7
**Healthy children**	14.3	1.9	12.8	14.2	15.8

	FTO gene region 1 methylation		FTO expression		PLAG1 expression	
**Blood pressure**	**Spearman's correlation coefficient r**	**p value**	**Spearman's correlation coefficient r**	**p value**	**Spearman's correlation coefficient r**	**p value**
Mean systolic blood pressure (mmHg)	0.212	0.319	0.196	0.359	0.141	0.512
Mean systolic blood pressure (percentile)	0.196	0.36	0.311	0.139	0.369	0.076
Mean diastolic blood pressure (mmHg)	0.132	0.537	-0.108	0.615	0.001	0.995
Mean diastolic blood pressure (percentile)	0.134	0.532	-0.332	0.743	0.177	0.409
Mean heart rate	0.033	0.879	0.095	0.657	0.209	0.327

Table S3. Correlation results of biochemical parameters with FTO gene methylation, expression and PLAG1 gene expression.

	FTO gene region 1 methylation		FTO expression		PLAG1 expression	
**Biochemical parameters**	**Spearman's correlation coefficient r**	**p value**	**Spearman's correlation coefficient r**	**p value**	**Spearman's correlation coefficient r**	**p value**
Total cholesterol (mmol/L)	0.057	0.79	0.094	0.664	-0.027	0.899
LDL cholesterol (mmol/L)	0.071	0.741	0.028	0.898	-0.008	0.969
HDL cholesterol (mmol/L)	-0.351	0.093	-0.008	0.969	-0.144	0.502
Free fatty acids – OGTT 60 min (mmol/L)	0.33	0.168	0.189	0.439	0.109	0.654
Free fatty acids - OGTT 120 min (mmol/L)	0.357	0.147	0.375	0.125	0.426	0.078
Glucose- OGTT 60 min (mmol/L)	-0.094	0.662	0.145	0.499	-0.03	0.889
Glucose- OGTT 120 min (mmol/L)	-0.217	0.308	0.052	0.809	0.142	0.509

3.3. Formatting oStudy design

This is example 1 of an equation:

OMICRON 10 V 2017r.

METHYLATION ANALYSIS

TARGET:

Pleiomorphic adenoma gene 1 (PLAG1)

Fat mass and obesity associated (FTO)

Methylation regions, based on Methyl-DIP (Nimblegene), tissue Whole Blood (source: Ensembl).

FTO (Chr16)

Region 1 (FTO – upstream)

53703684-53703899

53703663 ACGCCAGCAGAACTCCAGGGCCAACTCCAGGGCCTTCTCCAGGCGGCAGAGCGGA CCCTA 53703722

53703723 GGACC**CCGG**CCCGCGCTGCAGTGGGGAGGGTCAGCAACCTCCACCCACCCTCATCCTCCC 53703782

53703783 CCATCCTC**CCGG**GTACTCACCGTGCCACTGGCCCT GCAGCTAGCTACCGTTGCTATAGCG 53703842

53703843 CCGACAGCGTGGCGGGCGGCTGGCCGAGAGGAGCACGGGAGAAACATGGCAGGCTCCCGT 53703902

Region 3 (FTO – methylated CpG site in various cancer cell lines – data from WGBS)

54054572 (CG)

54054423 TTCCTGGGCAGGAAGGAGAGAATAGAGCCAAGCTGATGGAATTAAAACTGCGTGATTCAG 54054482

54054483 CCGGTATAAGACTGGAAATCTTTGTTACTGCCATGATGGTAATACCTTGTTATTAGTGCC 54054542

54054543 TCTGTCTTCGCTGAGCTGAGAAACCTC**C CG G**TGTGTTTTTGGCCATCAAAACTACTTGCC 54054602

54054603 ATTGTCACCGGTAGTCCAGATTTGTAAGGCTAAAGTGCTGAGGACAATTAGTAATTGGTG 54054662

54054663 ACTGGCTGCTATGATGTATAGTTGCTTAGCAACGGTGCCTGAATTAAGTCTCTGGAAGTG 54054722

Region 1 (PLAG1 exon1)

56211059-56211208

56211264 **ACAATGGCTGCTGGAAAGAGGCGTAAGGAAACAATTTCCAGGCCCGCCGCGTCCAGCCCG** 56211205

56211204 **AAATA TGAGAAAAAAATTATTAGAAATTCCGCGGGCGGTGTAGAGGCGGCGGACGGGCCG** 56211145

56211144 **GAGGGAGGATGTTAAAGCCCCGCG**GTGAGTTCT**CCGG**GGGT**CCGG**GGCGGCGGCGAGGCG 56211085

56211084 TTTAGCGGGAGAAATATCAGGGTTAT TTAAATTATGGGACTAGCCGAGGGGGCAGAGGAG 56211025
